# Chemsex as a Diagnostic Challenge: Toward Recognition in ICD-12 and Integrated Treatment Approaches—A Narrative Review

**DOI:** 10.3390/jcm14176275

**Published:** 2025-09-05

**Authors:** Justyna Śniadach, Wiktor Orlof, Justyna Sołowiej-Chmiel, Aleksandra Kicman, Sylwia Szymkowiak, Napoleon Waszkiewicz

**Affiliations:** 1 Department of Psychiatry, The Faculty of Medicine, Medical University of Białystok, 15-272 Białystok, Poland; 2 HCP Medical Center in Poznań, 61-485 Poznań, Poland; szymkowiaksylwia@gmail.com

**Keywords:** chemsex, sexuality, ICD-10, ICD-11, mixed addiction, substance use disorders, therapeutic strategies, compulsive sexual behavior disorder (CSBD)

## Abstract

Chemsex is a phenomenon involving the intentional use of psychoactive substances before or during sexual activity, especially among men who have sex with men (MSM). It is associated with various health risks, including substance dependence, risky sexual behaviors, and both mental and somatic disorders. Despite its growing prevalence and public health relevance, chemsex lacks a clear definition and is not recognized as a distinct diagnostic entity. This narrative review synthesizes clinical, epidemiological, and technological evidence on chemsex; argues for its classification as a form of mixed addiction; and preliminarily proposes diagnostic criteria for a potential entity in the International Classification of Diseases, 12th Revision (ICD-12). This paper highlights key psychotropic substances used in chemsex, patterns of use, and their neurobiological, psychological, and behavioral consequences. It explores the relationship between chemsex and compulsive sexual behavior disorder (CSBD), current diagnostic frameworks (ICD-10 and ICD-11), and challenges in clinical practice. Therapeutic strategies discussed include cognitive behavioral therapy (CBT), digital interventions, and emerging applications of artificial intelligence (AI) in prevention and treatment. Attention is also given to epidemiological trends, sociocultural influences, and barriers to seeking help. This review concludes by identifying research gaps and advocating for a more integrated, multidimensional approach to classifying and treating chemsex-related syndromes.

## 1. Introduction

Chemsex is a phenomenon involving the conscious use of psychoactive substances to intensify or prolong sexual sensations. Initially, it was mainly observed among men who have sex with men (MSM). However, a review of the scientific literature indicates that the phenomenon of chemsex is not limited to the MSM population but should also be considered in relation to the general population [1,2]. In recent years, there has been a growing interest in chemsex among heterosexual men and women, especially in party and club subcultures, where the use of psychoactive substances in sexual activity is an important part of the lifestyle [3].

The most common drugs in chemsex include methamphetamine, γ-hydroxybutyric acid (GHB), γ-butyrolactone (GBL), mephedrone and other cathinones, cocaine, and ketamine. Alkyl nitrites, so-called poppers, 3,4-methylenedioxymethamphetamine (MDMA), and phosphodiesterase type 5 (PDE-5) inhibitors, used to treat erectile dysfunction, are also frequently used. The use of multiple substances at the same time is common, and the specific combination may vary depending on region, availability, and the social and cultural context in which sexual activity takes place [4].

The effects of these substances are not limited to enhancing sexual sensations—these drugs increase the duration of sexual activity, enhance euphoria and increase feelings of connectedness while lowering anxiety and inhibition, which promotes more risky sexual behavior, such as unprotected sex, with multiple partners, or in prolonged sex sessions [3].

An important aspect of chemsex is also its psychosocial conditions. This phenomenon is often a response to stress, traumatic experiences, or pressure, related to cultural norms regarding sexuality [3]. There is also increasing attention to the fact that chemsex is a strategy for regulating emotions, including reducing anxiety or stress, which can lead to addiction and mental deterioration [4].

The rise in popularity of dating apps, based on geolocation technology, has significantly facilitated the establishment of contacts with potential sexual partners and the acquisition of substances, which has contributed to the dynamic development of chemsex [5]. Although this phenomenon is still most commonly observed among MSM, the literature indicates an increasing number of cases, including among women and heterosexuals, which underscores the need for a more thorough analysis of this phenomenon in different social groups [6].

Developments in digital technologies are opening up new opportunities for chemsex therapy, particularly in the context of artificial intelligence (AI)-based tools. Innovative technologies make it possible to monitor the behavioral patterns of chemsex-affected individuals, predict the risk of relapse, and provide personalized therapeutic interventions, which play a key role in the treatment of chemsex-related disorders, supporting both clinical interventions and prevention efforts [7].

Although chemsex is not currently recognized as a distinct diagnostic entity in The International Classification of Diseases (ICD)—ICD-11 classification, its complex nature—combining the misuse of psychoactive substances with compulsive sexual behaviors—may justify considering it a form of mixed addiction. From a clinical perspective, chemsex often involves co-occurring substance use disorder (SUD) and compulsive sexual behavior disorder, which aligns with existing diagnostic categories 6C_40_ and 6C_72_ in the ICD-11. In light of its increasing clinical and public health significance, there is a growing rationale to propose the inclusion of chemsex as a separate diagnostic category in the upcoming ICD-12 revision. Such classification could support more precise diagnosis, tailored treatment strategies, and improved health system coding and reporting.

## 2. Materials and Methods

This paper uses a narrative methodology, based on a systematic search of the literature on chemsex and its health, social, and technological consequences. The narrative review was conducted based on the literature search in databases including PubMed, Scopus, Google Scholar, and Web of Science, as well as other available sources. The following keywords were used in the search process: *chemsex*, substances such as *methamphetamine, GHB* (γ-hydroxybutyric acid), *GBL* (γ-butyrolactone), *mephedrone, ketamine, MDMA* (3,4-methylenedioxymethamphetamine, commonly known as ecstasy), *cocaine, poppers* (alkyl nitrites), *phosphodiesterase type 5 (PDE-5) inhibitors* such as *sildenafil, tadalafil, and vardenafil, 1,4-butanodiol, fentanyl* and other *opioids, kratom, sodium nitroprusside*, *α-PVP* (alpha-pyrrolidinopentiophenone), and *UR-144* (a synthetic cannabinoid), *sexuality, ICD-10, ICD-11, mixed addiction, substance use disorders, therapeutic strategies, compulsive sexual behavior disorder* (CSBD).

The exact search strings were constructed using Boolean operators (AND/OR) and adapted to the requirements of each database. Examples are presented below:

- PubMed: (“chemsex” OR “party and play” OR “PnP” OR “slamsex” OR “sex under the influence” OR “sexualized drug use”) AND (“methamphetamine” OR “crystal meth” OR “ice” OR “tina” OR “GHB” OR “γ-hydroxybutyric acid” OR “liquid ecstasy” OR “GBL” OR “γ-butyrolactone” OR “mephedrone” OR “4-MMC” OR “ketamine” OR “special K” OR “MDMA” OR “ecstasy” OR “cocaine” OR “crack” OR “poppers” OR “alkyl nitrites” OR “amyl nitrite” OR “PDE-5 inhibitors” OR “sildenafil” OR “tadalafil” OR “vardenafil” OR “Viagra” OR “Cialis” OR “Levitra” OR “1,4-butanediol” OR “BDO” OR “fentanyl” OR “opioids” OR “kratom” OR “Mitragyna speciosa” OR “sodium nitroprusside” OR “α-PVP” OR “flakka” OR “bath salts” OR “UR-144” OR “synthetic cannabinoids”) AND (“addiction” OR “substance use disorder” OR “SUD” OR “mixed addiction” OR “dual diagnosis” OR “compulsive sexual behaviour disorder” OR “CSBD” OR “sexual compulsivity” OR “problematic sexual behaviour” OR “ICD-10” OR “ICD-11” OR “ICD-12 draft”) AND (“treatment” OR “therapeutic strategies” OR “psychotherapy” OR “cognitive-behavioural therapy” OR “CBT” OR “harm reduction” OR “telemedicine” OR “eHealth” OR “mobile applications” OR “apps” OR “AI” OR “artificial intelligence”),
- Scopus: TITLE-ABS-KEY (“chemsex” OR “party and play” OR “PnP” OR “slamsex” OR “sexualized drug use”) AND TITLE-ABS-KEY (“methamphetamine” OR “crystal meth” OR “ice” OR “tina” OR “GHB” OR “GBL” OR “mephedrone” OR “ketamine” OR “MDMA” OR “cocaine” OR “poppers” OR “PDE-5 inhibitors” OR “sildenafil” OR “tadalafil” OR “vardenafil” OR “opioids” OR “fentanyl” OR “kratom” OR “α-PVP” OR “flakka” OR “UR-144”) AND TITLE-ABS-KEY (“addiction” OR “substance use disorder” OR “SUD” OR “mixed addiction” OR “compulsive sexual behaviour disorder” OR “CSBD” OR “ICD-10” OR “ICD-11”) AND TITLE-ABS-KEY (“therapy” OR “treatment” OR “telemedicine” OR “artificial intelligence”),
- Web of Science: TS = (“chemsex” OR “party and play” OR “PnP” OR “slamsex”) AND TS = (“methamphetamine” OR “GHB” OR “GBL” OR “mephedrone” OR “ketamine” OR “MDMA” OR “cocaine” OR “poppers” OR “PDE-5 inhibitors” OR “fentanyl” OR “kratom” OR “α-PVP” OR “synthetic cannabinoids”) AND TS = (“addiction” OR “substance use disorder” OR “SUD” OR “mixed addiction” OR “compulsive sexual behaviour disorder” OR “CSBD” OR “ICD-10” OR “ICD-11”) AND TS = (“treatment” OR “telemedicine” OR “mobile apps” OR “AI”),
- Google Scholar: searches combined (“chemsex” OR “party and play” OR “slamsex” OR “sexualized drug use”) with terms for substances (“methamphetamine”, “GHB”, “GBL”, “mephedrone”, “ketamine”, “MDMA”, “cocaine”, “poppers”, “fentanyl”, “kratom”, “α-PVP”, “synthetic cannabinoids”), diagnostic frameworks (“ICD-10”, “ICD-11”, “mixed addiction”, “substance use disorders”, “compulsive sexual behaviour disorder”, “CSBD”), and treatment approaches (“psychotherapy”, “therapeutic strategies”, “harm reduction”, “telemedicine”, “AI”, “mobile applications”).

The studies included in the review comprise peer-reviewed publications available between 2015 and 2025, ensuring that the results are up-to-date and aligned with the current state of knowledge.

The analysis focuses on clinical, technological, and psychological aspects of chemsex, with particular emphasis on the role of modern technology in addiction treatment, including mobile apps, telemedicine platforms, and AI tools.

### 2.1. Inclusion Criteria

Studies meeting the following criteria were considered for analysis:

- Publications from 2015–2025 that provide up-to-date data on chemsex and its health consequences.
- Studies on psychosocial aspects of chemsex, therapeutic technologies, and the effectiveness of modern treatment methods.
- Studies analyzing the interaction of psychoactive substances with mental and sexual health.
- Articles describing therapeutic strategies, including the use of telemedicine and AI tools in monitoring user behavior patterns.

### 2.2. Exclusion Criteria

The following were excluded from the review:

- Publications not subject to scientific peer review and opinion articles that did not provide empirical data.
- Studies focusing only on general social aspects of chemsex, without reference to health effects.
- In vitro laboratory work that did not provide direct evidence of the effects of chemsex on the human body.
- Publications with low methodological transparency and showing a high risk of bias.

To ensure clarity and transparency, the criteria for study selection were organized into two main categories: inclusion and exclusion. Presenting them in tabular form allows the reader to quickly identify the scope of studies considered eligible and those excluded. The inclusion and exclusion criteria applied in this review are summarized in Table 1 for improved readability.

### 2.3. Article Selection and Error Minimization

The initial analysis included 370 articles, which were independently evaluated for their compliance with the research objectives. After verification of titles and abstracts, 295 full texts were selected that met the criteria for preliminary methodological evaluation. In the next stage, they were analyzed in detail, taking into account their content value, methodological value and compliance with the study objectives.

**Figure 1:**
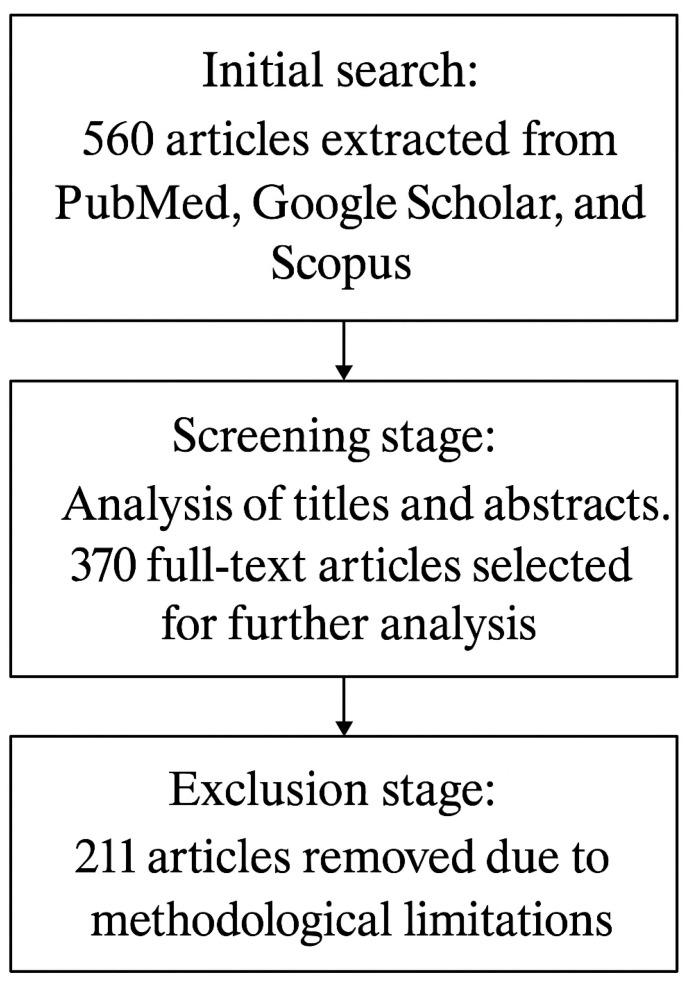
Flowchart illustrating the article selection process.

The final selection was based on a structured selection process designed to ensure objectivity and methodological soundness (Figure 1). As a result of this process, 211 articles were rejected, mainly due to lack of scientific peer review, poor methodological quality, limited coverage of the topic, or high risk of bias.

To minimize the risk of bias, the article selection process was conducted by two independent experts with experience in addiction and public health research. In the case of discrepancies, decisions were made after consultation with a third reviewer, who acted as a scientific arbiter. In the end, 84 articles were included in the review that provided the most relevant and high-quality evidence on chemsex, its health effects, and the use of modern technology in addiction treatment (Figure 1). Substances used during chemsex have a variety of pharmacological properties.

Although this work was based on a systematic search strategy, it should be emphasized that the present study is a narrative review and therefore has certain inherent limitations. In contrast to systematic reviews, no formal and comprehensive quality assessment of each included study was performed. The synthesis of evidence was qualitative and descriptive, aimed at integrating clinical, epidemiological, and technological perspectives rather than providing a quantitative meta-analysis. As a result, the conclusions presented should be interpreted in light of these methodological constraints.

## 3. Chemsex: Origins of the Phenomenon, Definitions, and Health Consequences: Findings from the Literature Review

“Chemsex,” literally translated, means sex under the influence of psychoactive drugs. However, around its definition, there are still debates among specialists regarding the question of the populations to which the term would apply, the substances taken, and how much of it is a new phenomenon and how much has been known for many centuries [8,9].

According to Stuart [10], chemsex should be considered more broadly than simply the use of drugs for sexual activity, since the phenomenon itself has a long history and takes place in many different populations; yet, the activity will not always fall within the term described. At the turn of the 20th century, in London, the term “chems” was used as slang for methamphetamine and GHB/GBL when communicating with dealers over the phone. At the time, methamphetamine was hard to come by, and its use was associated with social ostracism, resulting in the creation of a community of men who, when meeting, would reach for the aforementioned substance. This group began to refer to itself as the “Chemsex Club,” and it is this name that forms the foundation of the phenomenon under analysis [10]. The phenomenon of chemsex itself was already observed at the turn of the 20th century but gained in popularity in 2006, when a higher prevalence of mephedrone was observed among the British population [10,11]. The term “chemsex” itself, on the other hand, began to function in the literature, thanks to a paper titled: “The chemsex study” [12,13,14,15].

Chemsex, as a social and health phenomenon, diversified in terms of culture, society, and availability of psychoactive substances. Although the practice is global in scope, the nomenclature used and the way it is carried out are subject to regional and cultural modifications. In Europe and parts of South Asia, the predominant term is “chemsex,” while in North America, Australia and New Zealand the term “party and play” is more commonly used. A specific and riskier form of this practice is so-called “slamsex,” which involves the intravenous injection of psychoactive substances during sexual activity, which significantly increases the risk of blood-borne infections, including human immunodeficiency virus (HIV) and hepatitis C virus (HCV) [13,14,15]. Chemsex is most commonly reported among MSM populations, but its prevalence varies, with estimates ranging from 3% to as high as 42%, depending on the population studied, the country, and local socio-cultural conditions. The highest rates are usually observed in large metropolitan areas and in communities characterized by high levels of anonymity and easy access to psychoactive substances [13].

### 3.1. The Diversity and Dynamics of the Development of the Chemsex Phenomenon

One of the key determinants of chemsex is the socio-cultural context. In large, anonymous cities, where cultural diversity is greater, chemsex can play an emancipatory role, giving participants the opportunity to experiment with sexual identity and break social norms. At the same time, it carries risks associated with substance abuse and engaging in risky sexual behavior [16,17,18].

The phenomenon of chemsex should also be considered in the broader context of social changes in the perception of sexuality. Modern sexual culture, based on the idea of freedom and consensually—according to the maxim “anything that suits both partners is allowed, as long as no one suffers from it”—does not always lead to greater satisfaction with sexual life. Sexual freedom does not necessarily translate into lasting satisfaction—the number of people reporting dissatisfaction with this sphere of life is increasing [19].

The reasons for this can be traced to several contemporary social phenomena that may indirectly promote engagement in chemsex:

- Social pressure and comparison—the media promote idealistic images of sexuality, leading to frustration and dissatisfaction;
- Increased expectations—greater freedom creates new norms and higher expectations, and failure to meet them generates disappointment;
- Lack of emotional connection—short-lived, casual sexual relationships without commitment can heighten feelings of emptiness;
- Stigma and guilt—conflicts between desires and cultural norms can exacerbate internal tensions;
- Self-esteem problems—low self-esteem is associated with difficulties in building satisfying relationships;
- Sexual burnout—frequent contact with stimuli can lead to fatigue and decreased pleasure;
- Fear of rejection—fear of being judged and disapproved influences the choice of less stable relationships.

All of the above factors may promote an escape into chemsex as an alternative means of achieving sexual and emotional gratification [20].

In addition, minority stress mechanisms, internal homophobia, or social pressure may contribute to the more frequent use of psychoactive substances. Studies indicate that personality aspects, such as conscientiousness, can be important predictors of behavior, associated with chemsex, and, in some cases, influence decisions regarding how to consume substances and the context of their use [21].

The increasing prevalence of digital technologies today, including dating apps that facilitate contact with potential partners and substance acquisition, significantly dictate the dynamics of behaviors associated with chemsex [22].

In the context of addiction, it is worth noting the neurobiological mechanisms involved in chemsex. As Waszkiewicz [23] points out, addictions to psychoactive substances often co-occur with behavioral addictions, such as compulsive sexual behavior. In the case of chemsex, there is a synergy between the two types of addiction, which makes the therapeutic process more difficult and requires an integrated treatment approach [23].

In some communities, chemsex is seen as a form of “wild self-care,” where psychoactive substances are treated as a tool for overcoming inhibitions, exploring identity, and coping with stress. This dimension, while important from an emotional perspective, can increase the risk of addiction and escalation of risky behavior in the long term [22].

### 3.2. Prevalence of Chemsex

The phenomenon of chemsex is increasingly the subject of epidemiological studies, particularly in the context of the MSM population, among whom it is most prevalent. One of the largest studies conducted in the United States, involving 30,294 MSM, found that 10.3% of respondents (3113 individuals) reported using psychoactive substances in a sexual context in the past 12 months [24]. Among these individuals, 65.1% reported using MDMA, 42.5% reported using methamphetamine, and 21.7% reported using GHB. Significantly, this study did not include the use of mephedrone or other synthetic cathinones, despite their prominent role in European chemsex contexts [24]. It was also noted that chemsex users in the U.S. were significantly younger than their counterparts in Europe—the majority were around 20–22 years old [25].

The latest data in Poland come from Agata Stola’s research, which the author presented in her doctoral dissertation titled “Psychosocial determinants of the chemsex phenomenon in Poland” [26]. The survey of MSM showed that about 11.9% of respondents had chemsex experiences, a figure slightly lower than the European average of 15.2%. The most commonly used substances in Poland were mephedrone (86.4%), GBL/GHB (68.2%), and other synthetic stimulants (45.5%). The place of practice of chemsex was most often private homes (49.5%) and own apartments (22.9%). The report also shows that 39.5% of Polish chemsex users were diagnosed with HIV infection, and among those with more than three years of psychoactive substance use, the percentage was already 53.3%. Among the most common reasons for engaging in chemsex, study participants cited the following: increasing the intensity of sexual intercourse (84.8%), intensifying pleasure (75.8%), and increasing the duration of sexual intercourse (73.4%) [26]. It is worth noting that research on chemsex in Poland has focused mainly on the MSM population, while data on heterosexuals remain limited. This makes it difficult to accurately estimate the true extent of the phenomenon in broader populations.

In Poland, the epidemiology of drug use—including in the sexual context—is systematically monitored by institutions such as the National Bureau for Drug Prevention, as well as international organizations, including the European Monitoring Center for Drugs and Drug Addiction. Although some qualitative studies and local reports suggest a growing interest in the use of psychoactive substances in a sexual context, including among heterosexuals, especially in club environments, there is still a need for more detailed quantitative and qualitative research taking into account diverse social groups [24].

Contemporary research indicates that as many as 99% of methamphetamine users and 75% of mephedrone users use these substances solely to facilitate sexual activity. GBL, though used less frequently, also finds use in a sexual context, with 5% of its users reporting such motivations. Of particular concern is the increase in the use of methamphetamine administered intravenously—the percentage rose from 20% in 2011 to 80% in 2013. Significantly, up to 70% of users of this form of drug use share needles and syringes, which drastically increases the risk of blood-borne infections such as HIV and HCV [10].

Epidemiological data also indicate that 75% of people who use psychoactive substances for sexual activity are HIV-positive, and of these, as many as 60% report not adhering to an antiretroviral therapy (ART) regimen during periods of drug use. Such behavior not only exacerbates health risks for the users themselves but also for their sexual partners and entire communities [27].

#### 3.2.1. Gender-Specific Patterns of Chemsex

Most epidemiological studies on chemsex have primarily focused on men who have sex with men (MSM) [6,12,13,25,28,29]. In these populations, the prevalence is consistently higher compared with the general population. Methamphetamine, mephedrone, and GHB/GBL are the most frequently reported substances [6,12,16,30,31]. Slamsex practices, involving the injection of stimulants during sexual encounters, are also more often described in this group and are strongly associated with increased risks of HIV and other sexually transmitted infections (STIs) [12,13,18,25,32]. MSM-specific social networks and the use of dating applications further facilitate the organization of chemsex sessions, which contributes to the higher frequency and intensity of substance use [16,31].

In contrast, women are less frequently represented in the available literature on chemsex. When described, their participation is often associated with recreational or club contexts rather than structured chemsex networks [6,33,34,35]. Despite the lower prevalence, the potential risks remain substantial. Women face particular vulnerability to sexual assault while under the influence of psychoactive substances, and a significant knowledge gap persists due to their underrepresentation in epidemiological research [33,34]. This lack of data makes it difficult to tailor harm reduction measures or clinical interventions specifically for women engaged in chemsex.

To better visualize these differences, Table 2 summarizes the main epidemiological characteristics of chemsex participants according to gender identity, with emphasis on MSM and women, while also indicating specific risks observed in transgender individuals.

#### 3.2.2. Chemsex in Transgender Populations

Transgender individuals represent a population at particularly high risk of chemsex-related harm. While most research has concentrated on MSM, emerging evidence shows that transgender people not only engage in chemsex but often present with distinct vulnerabilities compared to both men and women [33,36,37]. Reported prevalence rates are higher than in cisgender populations, and patterns of use are characterized by frequent polysubstance consumption and more severe health consequences [37].

Compared to MSM, transgender individuals often experience a greater burden of psychological distress and minority stress, which can intensify chemsex behaviors and exacerbate harmful outcomes [33,37]. Unlike women, who are more often described in recreational or club contexts, transgender people are disproportionately exposed to marginalization, stigma, and discrimination, which compound the risks associated with substance use. These psychosocial stressors not only increase vulnerability to chemsex initiation but also hinder access to appropriate healthcare and support services [33,36].

The impact of drug use in transgender populations extends beyond physical health outcomes such as higher rates of HIV and STIs. Mental health consequences—including depression, anxiety, and social isolation—are reported to be more severe in this group, reflecting the combined effects of substance use and structural discrimination [33,36,37]. Taken together, these findings underscore the urgent need for targeted harm reduction strategies and inclusive treatment approaches that specifically address the realities of transgender individuals engaged in chemsex.

### 3.3. The Most Commonly Used Substances in Chemsex and Their Effects

Substances used during chemsex have a variety of pharmacological properties. Chemsex involves the use of various psychoactive substances to intensify sexual sensations, reduce inhibitions, and prolong activity [25,26]. The practice of chemsex involves the use of both stimulants (e.g., methamphetamine, mephedrone, cocaine) and depressants (e.g., GHB, GBL), often used concurrently or sequentially, depending on the desired effect, availability of the substance, and social context [27].

The literature distinguishes the three most popular substances among users as primary to the so-called “classic chemsex”: methamphetamine, GHB/GBL, and mephedrone (see Table 3). However, systematic studies indicate that there are regional differences in psychoactive substance preferences. For example, in Europe, chemsex practices often include MDMA, ketamine, and cocaine, which, while less frequently associated with the typical chemsex profile, play a significant role in certain socio-cultural contexts [38].

Methamphetamine is a powerful stimulant with psychoactive effects that plays an important role in the context of chemsex. Its use is associated with intense euphoria, arousal, and a significant increase in libido, making it particularly attractive to those seeking increased intensity of sexual experience. The substance improves sexual stamina, reduces inhibitions, and intensifies sexual adventurousness. The effect of methamphetamine on the central nervous system is to increase the release of dopamine, resulting in strong feelings of pleasure but at the same time contributing to the rapid depletion of neurotransmitter reserves [33,39]. The result is an increased sense of self-confidence and a willingness to engage in risky sexual behavior. Methamphetamine prolongs the duration of sexual activity, which is perceived by users as beneficial, but at the same time is associated with a high physical burden on the body. Sustained arousal over a long period of time can lead to dehydration, overheating and energy depletion. In addition, increased sexual intensity and reduced behavioral control significantly increase the risk of sexually transmitted infections (STIs), including HIV and HCV. Long-term methamphetamine use is associated with permanent neurotoxic changes that lead to deterioration of cognitive functions such as memory, attention, and impulse control and contribute to the escalation of psychiatric disorders. Abusers of this substance are particularly prone to anxiety, depression, and psychotic episodes. In addition, with prolonged use, sexual dysfunctions such as erectile problems in men and lube disorders in women are observed [40,41].

Methamphetamine has a strong addictive potential—especially psychological—as the intensity of the effects experienced leads to the rapid development of a compulsion to use again. Due to the frequent co-occurrence of chemical dependence with behavioral addictions, the therapeutic process is extremely difficult [42].

GHB and its precursor GBL are substances with depressant potential, strongly affecting the central nervous system, and are commonly used in the context of chemsex. Their mechanism of action is based on the activation of γ-aminobutyric acid (GABA) receptors, leading to sedation, relaxation, feelings of euphoria, and reduced anxiety [43]. Such effects promote increased openness to social and sexual contact, making these substances particularly attractive during sexual events and intimate encounters. An increase in perceived closeness and a reduction in inhibitions can lead to engaging in risky sexual behavior, such as unprotected sex. GHB is the pharmacologically active form, while GBL undergoes enzymatic conversion in the liver to GHB through a hydrolysis reaction. Thus, in this aspect, GBL is a precursor of GHB (pro-drug), and its activity depends on the rate of conversion to the active metabolite. Similarly, 1,4-butanediol acts similarly, converting to GHB via the enzymes alcohol and aldehyde dehydrogenase. Both GBL and 1,4-butanediol are more toxic to the body than GHB alone [44].

In the context of chemsex, GHB/GBL are mainly used to intensify sexual sensations, prolong intercourse, and reduce social and sexual anxiety. Their use helps to increase openness with partners and reduce emotional barriers. However, long-term use of these substances can lead to dependence, both psychological and physical, which is associated with the occurrence of withdrawal syndrome, most often manifested in the form of seizures, anxiety, insomnia, and cardiac arrhythmias [45].

Similarly, 1,4-butanediol (1,4-BD), a less commonly discussed GHB precursor, is converted into GHB in the body through the action of alcohol dehydrogenase and aldehyde dehydrogenase enzymes. Its delayed onset and unpredictable pharmacokinetics can pose significant risks, including unintentional overdose [17].

Mephedrone, a member of the synthetic cathinone group, is a psychoactive substance with powerful stimulant effects. It acts as a reuptake inhibitor of serotonin, dopamine, and norepinephrine, leading to increased euphoria, improved mood, and increased energy, as well as an increased need for social contact [46]. Due to its properties, mephedrone is readily used in sexual activity. The substance intensifies sexual desire (increased libido) and boosts self-confidence, prompting risky sexual behavior. Users declare an intensification of sensual and emotional sensations and a delay in orgasm, allowing for prolonged sexual intercourse. These effects may be perceived as beneficial, but they carry significant psychophysical health risks. Erectile problems and hypolibidemia can occur after withdrawal from the substance following prolonged use. After discontinuation of mephedrone, there is often a so-called “descent,” i.e., a rapid deterioration of mood, lowering of mood, and increased irritability. Increased sexual intercourse under the influence of this substance is also associated with a higher risk of STIs. Mephedrone is a derivative of cathinone, a naturally occurring psychoactive substance found in the leaves of the khat shrub (Catha edulis), traditionally chewed in East African and Arabian Peninsula countries. In synthetic form, mephedrone was for a time sold legally as “bath salts.” Although the metabolites of mephedrone do not exhibit neurotoxicity, the substance itself can lead to severe psychological dependence [34]. Even small doses can cause persistent difficulties with attention and memory, as well as a range of side effects such as nosebleeds, cardiac arrhythmias, hallucinations, panic attacks, anxiety, paranoia, delusions, and hyperactivity [30].

Ketamine is a synthetic dissociative that has gained popularity among chemsex participants for its properties that enable detachment, relaxation and pain suppression. The substance acts on N-methyl-D-aspartate (NMDA) receptors in the central nervous system, leading to a sense of depersonalization and distorted perception, both of time and space. In the practice of chemsex, it is used to reduce stress, increase sexual desire, and relieve perceived pain, especially during prolonged sexual activity. Ketamine takes effect about 5–10 min after taking it, and the effects last for 30–60 min. The onset and duration of action depend on the route of administration—intravenous administration leads to a faster onset and shorter duration, while intranasal and oral routes result in a slower and more prolonged effect [47]

Its biological half-life is between 2 and 4 h, making it a relatively short-acting agent, often combined with other psychoactive substances. However, such combinations carry a high risk of pharmacological interactions and serious health complications [48]. The results of previous studies indicate that ketamine significantly reduces the risk of suicide and exhibits antidepressant and anti-anxiety effects, which may also contribute to its treatment as a coping strategy for mental distress [49]. However, in addition to its euphoric and anxiety-lowering effects, its use is associated with a number of side effects. Users often experience attention problems—ketamine can make it difficult to focus on a partner or the sexual act itself, leading to distraction. Another frequently observed side effect is difficulty achieving orgasm in both men and women. Long-term use can lead to increased tolerance, psychological dependence, and mood disorders, as well as cognitive impairment and psychotic episodes [50].

MDMA, also known as ecstasy, is a substance with stimulant and empathogenic effects. It is gaining its popularity in chemsex due to its ability to intensify emotional and sensory experiences. It works by increasing the synaptic concentration of serotonin, dopamine, and norepinephrine, leading to euphoria, energy, an increased sense of happiness, and a desire for social contact and interpersonal closeness. MDMA intensifies feelings of empathy and trust, which increases feelings of connection with a partner. Proponents of the substance report feeling increased sensitivity to touch and intensification of sensory experience. At the same time, despite increased stimulation, MDMA can make it difficult to achieve orgasm, which is particularly noticeable in men [51]. MDMA’s effects usually last from 3 to 6 h, with a biological half-life of 7–9 h. The effects appear 30–60 min after use. Negative effects of MDMA use include jaw tightness, insomnia, increased blood pressure, nausea, and dehydration. Once the effects are over, there is a so-called “downhill slide”—a sudden drop in mood, fatigue, irritability, and depression that can last up to 48 h. MDMA is also sometimes combined with other psychoactive substances, increasing the risk of health complications and pharmacological interactions [52].

**Table 3:** Pharmacological properties of substances used in chemsex based on [17,28,33,36,47,52,53,54,55,56,57,58,59].

Substance	Action	Potential Risks
**Methamphetamine**	Intense euphoria, sexual arousal, increased libido, impulsivity, prolonged sexual activity	Neuronal damage, psychotic episodes, depression, addiction, erectile dysfunction, risky sexual intercourse
**GHB/GBL**	Euphoria, relaxation, reduction of anxiety, increased openness, intensification of sensations	Addiction, respiratory depression, loss of consciousness, withdrawal symptoms, interaction with alcohol
**Mephedrone**	Euphoria, increased energy, improved mood, sexual stimulation, increased self-confidence	Addiction, cardiac arrhythmia, anxiety, paranoia, hallucinations, erectile and orgasmic problems
**Ketamine**	Dissociation, intensification of sensations, detachment from reality, muscle relaxation	Psychotic states, depression, concentration and orgasmic problems, pharmacologic interactions
**MDMA (ecstasy)**	Euphoria, empathy, energy, increased sensitivity, intensification of closeness	Difficulty reaching orgasm, dehydration, overheating
**Cocaine**	Energy, stimulation, euphoria, sociability, mood enhancement	Addiction, hypertension, anxiety, depression, rapid drop in mood after action
**Poppers (nitrites)**	Short-term euphoria, smooth muscle relaxation, intensification of sexual sensations	Cardiovascular complications, depression, drops in blood pressure, dangerous interactions
**PDE-5 inhibitors (sildenafil, tadalafil, vardenafil)**	Increased blood flow, improved erection, prolonged intercourse	Hypotension, interaction with poppers, headaches, visual disturbances
**1,4-Butanodiol**	Calming effect, increases feelings of closeness	Respiratory depression, withdrawal symptoms
**Fentanyl and other opioids**	Intensification of sensations, reduction of pain	Respiratory depression, high risk of addiction
**Kratom**	Stimulant at low doses, sedative/analgesic at higher doses	Dependence, withdrawal, hepatotoxicity

Cocaine is a powerful central nervous system stimulant that increases dopamine levels in the brain, leading to euphoria, increased energy levels, confidence, and sociability. In the context of chemsex, it is used primarily for its rapid stimulant effect and its ability to enhance sexual sensations. Cocaine’s effects are intense but short-lived—usually lasting from a few minutes to an hour. After they subside, feelings of fatigue, irritability, and a drop in mood are common, which can prompt repeated use. Regular cocaine use increases the risk of addiction, hypertension, cardiac arrhythmias, and mental and emotional problems [53].

Alkyl nitrates (poppers) are one of the most commonly used groups of substances in chemsex, although they are not formally classified as drugs. Inhaled vapors of compounds such as amyl, butyl, or isobutyl nitrite lead to the immediate vasodilation of blood vessels and relaxation of smooth muscles, especially in the anal and vaginal areas. The effect is a brief feeling of euphoria, warmth, intensification of sexual sensations, and accelerated heartbeat. In sexual practice, poppers are used to facilitate penetration, reduce pain, and increase the pleasure of physical contact. Their effects appear almost immediately—just a few seconds after inhalation—but are short-lived. Depending on the chemical compound, the duration of the effects usually ranges from 30 s to 3 min, with a possible residual effect lasting for several minutes, in the form of relaxation and a feeling of warmth. Amyl nitrite (1–2 min) and butyl nitrite (up to 2 min) have the shortest effect, while isobutyl nitrite (2–3 min) has a slightly longer effect [54]. It is worth noting that poppers are often used concurrently with other psychoactive substances, which increases the risk of health complications. Combining them with PDE-5 drugs such as sildenafil or other psychoactive agents can lead to cardiovascular complications such as hypotension and circulatory collapse. In the long term, poppers use is also associated with a risk of depression, anxiety, and psychotic episodes [28]. In addition, an increase in risky sexual behavior following their use may promote infections, including HIV [55].

PDE-5 inhibitors, such as sildenafil, tadalafil, and vardenafil, are drugs primarily used to treat erectile dysfunction. Their action is to increase blood flow to the genitals by inhibiting the PDE-5 enzyme, leading to improved erections, increased sexual performance and longer duration of sexual intercourse. In the context of chemsex, these drugs are often used in combination with other psychoactive substances to maintain erections, despite the concomitant effects of agents that impair libido and/or sexual performance. Combining PDE-5 inhibitors with other psychoactive substances, especially poppers (alkyl nitrites), carries serious health risks, causing, among other things, rapid drops in blood pressure that can lead to unconsciousness, heart attack, and even death [56]. With tadalafil and sildenafil, a break of at least 24–48 h is required to avoid life-threatening interactions [57].

Additional studies highlight that the widespread use of PDE-5 inhibitors in chemsex is linked to both increased duration of intercourse and engagement in high-risk sexual practices, which further exacerbates the risk of STIs, dependency, and cardiovascular side effects [58,59,60].

Although not traditionally associated with chemsex, fentanyl and other opioids are sometimes used to enhance physical sensations and reduce pain during prolonged sexual activity. Their potent sedative and analgesic effects may be sought after by some users; however, opioids carry a high risk of respiratory depression, especially when combined with depressants such as GHB or alcohol, increasing the likelihood of overdose and fatal outcomes [31]. Additionally, the delayed effects of some opioids may impair the user’s ability to accurately dose or gauge impairment, further contributing to hazardous scenarios [59].

Kratom (Mitragyna speciosa) is a psychoactive plant increasingly reported among chemsex users [61]. In low doses, it acts as a stimulant, enhancing energy and sociability; in higher doses, it exhibits sedative and analgesic properties. Some users may employ kratom as a perceived “natural” alternative to opioids or stimulants. However, chronic use has been associated with dependence, withdrawal symptoms, liver toxicity, and cognitive impairment. Its unregulated status in many countries also leads to variability in potency and adulteration, increasing health risks [60].

### 3.4. Dosage, Patterns of Use, and Related Risks

Patterns of substance use in chemsex are considerably varied, ranging from occasional, event-based use to more regular and continuous patterns that carry higher health risks. Typical doses are difficult to standardize due to the variability of illicit substances; however, some general patterns are consistently reported. For example, methamphetamine is most often consumed in doses of 50–200 mg per session, with effects lasting up to 12 h. Repeated dosing during a single chemsex event is common and significantly increases the risk of neurotoxicity, insomnia, and psychotic symptoms [33,38,39,40,41].

GHB and GBL are typically taken in small liquid doses of 1–2 mL, repeated every 1–3 h to maintain the desired effects. Due to their narrow therapeutic index, the difference between a recreational and a toxic dose is minimal, creating a high risk of overdose, respiratory depression, and life-threatening drug–drug interactions, particularly with alcohol or opioids [43,44,45].

Mephedrone is usually consumed in doses of 100–250 mg orally or intranasally, often redosed multiple times throughout long sexual sessions. Continuous use is strongly associated with dependence, cardiovascular strain, and severe withdrawal symptoms [30,34,46]. Ketamine, most commonly administered intranasally in doses of 30–100 mg, has a shorter duration of effect (30–60 min) and is frequently combined with stimulants. Such combinations substantially increase the risk of cognitive impairment and unpredictable pharmacological interactions [47,48].

MDMA is generally ingested orally in single doses of 75–125 mg, with effects lasting 3–6 h. Occasional use is associated mainly with acute adverse effects such as hyperthermia and dehydration, while repeated use within a single event or across weekends increases the risk of serotonin depletion, depression, and prolonged cognitive impairment [51,52].

Poppers, inhaled directly from small vials, act within seconds and last only 1–3 min. Although frequently used continuously during sexual activity, they carry significant cardiovascular risks, especially when combined with PDE-5 inhibitors such as sildenafil or tadalafil [28,54,55,56,57]. PDE-5 inhibitors themselves are usually consumed at therapeutic doses (sildenafil 25–100 mg, tadalafil 5–20 mg), but their combination with stimulants or nitrites markedly increases the risk of hypotension, arrhythmias, and sudden cardiac events [56,57,58].

In summary, occasional use is mainly associated with acute intoxication, risky sexual behavior, and short-term mental health deterioration, whereas continuous or high-frequency use contributes to long-term complications, including dependence, neurotoxicity, and cumulative drug–drug interactions. These distinctions underline the importance of assessing not only the substances themselves but also their dosage, frequency, and context of use when evaluating the health risks associated with chemsex [43,44,45,47,57,62,63].

### 3.5. Sociocultural Context of Chemsex

Studies indicate that many MSM involved in chemsex use three or more substances before or during sexual intercourse, and these are not always classic chemsex substances. Stevens et al. [32] noted that among drug users associated with chemsex, alcohol was the most commonly consumed substance [21]. The circumstances and venues in which MSM practice chemsex are also important—anonymous sessions organized most often online, via geolocated dating apps or social media, semi-closed events within close social networks, spontaneous sexual encounters referred to as “chillouts” or “chill-sex,” and spaces offering sex-on-premises venues, such as saunas or darkrooms. In these spaces, diverse and intense sexual practices are common, such as anal sex, oral sex, group sex, fisting, and the use of erotic toys [21].

One variation of chemsex is known as slamsex, which is sexual activity undertaken under the influence of psychoactive substances taken intravenously, as opposed to chemsex, where supply is by oral, intranasal, or rectal routes [64].

Substances used in chemsex can indirectly lead to uncontrolled and impulsive behavior, as well as engaging in risky sexual behavior, such as intercourse without a condom. This increases the likelihood of contracting STIs, including HIV. Slamsex practitioners are more likely to be diagnosed with sexually transmitted diseases and mental health problems [21]. Regular drug use in the context of chemsex can lead to addiction or abuse, which negatively affects physical health, mental health, and overall emotional well-being. It can also result in anxiety, depression, and psychotic symptoms, which sometimes persist even after the effects of the substance have subsided. Chemsex practitioners may have difficulty experiencing satisfaction with their sex lives without substance use. Many former chemsex users report difficulty enjoying sex, with some finding drug-free intercourse very difficult or even impossible [21,64].

### 3.6. New Substances Used in Chemsex

With the growing popularity of chemsex, new psychoactive substances (NPSs) are emerging that can significantly affect users’ experiences (Table 3). The following are characteristics of selected NPSs that can affect the sexual experience:

1. Psychedelics (LSD, psilocybin, DMT, mescaline, MDMA): These substances can put one in a state of intense perception and alter their perception of sexual sensation. Although not typical of chemsex, their use in this context can lead to unpredictable consequences [58].
2. Sodium nitroprusside: It is used in medical settings, but its use in chemsex is gaining popularity, due to its strong vasodilator effect. It can lead to dangerous drops in blood pressure, especially when combined with other stimulants [65].
3. α-PVP: This is a substance functioning under the name “Flakka” or “zombie drug.” It is an organic chemical compound, a pyrrolidine derivative of valerophenone, showing similarity to cathinone (an amphetamine derivative). It has stimulant, empathogenic, and euphoric properties. It can come in powdered form (resembles fine crystals, bath salt, or grit, which is used for aquariums). It can be taken in tablet form, injected intravenously, smoked, or snorted through the nose. After taking it, feelings of euphoria, increased alertness, and insomnia appear but also psychomotor agitation. Its use can also result in increased aggressiveness and greater resistance to pain [62,63,66,67].
4. UR-144: This is a synthetic cannabinoid that was detected in a number of “legal highs” seized from the drug market in 2012. It has gained popularity as a marijuana alternative in countries where it has not been controlled. It is most often mixed with tobacco or herbs and smoked, to produce effects similar to marijuana. It can also be taken orally or vaporized and inhaled. The effects reported by users typically begin 0.5 to 2 min after ingestion, peak after 3 to 5 min, and end after 1 to 2 h; in situations of higher supply, the effects can last even up to 4 h. The substance causes relaxation, improved mood, and cheerfulness but also delayed pupillary response, slurred speech, and even unconsciousness [68].

The characteristics of selected NPSs used in chemsex are presented in Table 4.

## 4. Chemsex: A New Diagnostic Challenge in Addiction Medicine

According to the ICD, 11th revision (ICD-11), chemsex can be recognized as a disorder resulting from the use of psychoactive substances. It meets the criteria for substance use disorder, such as a strong desire to use, loss of control over use, continued use despite harm, and withdrawal symptoms [69]. The use of psychoactive substances in chemsex primarily serves to intensify sexual sensations and, over time, may lead to substance dependence [70].

Importantly, chemsex differs from sex addiction. While sex addiction is classified in the ICD-11 as an impulse control disorder involving compulsive sexual urges that impair functioning, chemsex involves the fusion of substance use and sexual behavior. Individuals engaging in chemsex often become dependent on substances to initiate or maintain sexual activity. This dependence on combined experiences presents challenges in therapy, where patients may find it difficult to engage in substance-free sex [71].

**Figure 2:**
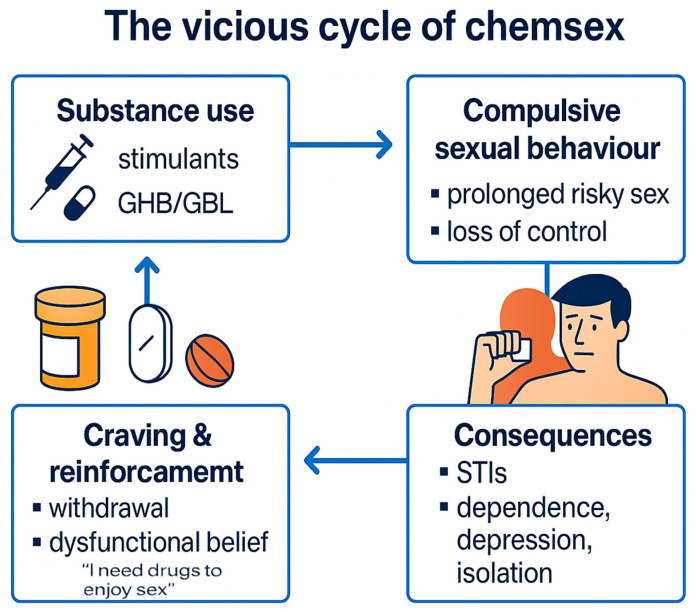
The vicious cycle of chemsex.

Furthermore, chemsex involves a dynamic interplay between substance use, compulsive sexual behavior, and their physical and psychosocial consequences. These factors reinforce one another through craving and dysfunctional beliefs, creating a vicious cycle that is illustrated in Figure 2.

Chemsex lies at the intersection of psychoactive substance addiction and sex addiction. While the ICD-11 defines substance addiction as compulsive use despite harm, and sex addiction as an impulse control disorder involving uncontrollable sexual urges [68], chemsex combines both dimensions.

Individuals practicing chemsex often report difficulty separating sexual activity from substance use, reinforcing both behavioral and chemical dependence. This dual pattern complicates therapy, as traditional treatment approaches may not adequately address both aspects simultaneously [69].

To address this complexity, dedicated treatment programs are necessary (see Figure 2 for a summary of key harmful effects). These should integrate pharmacological treatment with psychotherapy, focusing on both substance use and sexual behavior. Such comprehensive programs could improve outcomes and reduce relapse risk [72].

Moreover, the lack of a distinct diagnostic category for chemsex in the ICD-11 hinders recognition and treatment. Recognizing chemsex as a separate clinical entity could support better understanding and more effective interventions [70].

### 4.1. Chemsex as a Mixed Addiction: Interplay of Substance Use and Compulsive Sexual Behavior

Chemsex should be considered a mixed addiction, as it combines features of both psychoactive substance dependence and behavioral addiction associated with sexual activity. Individuals engaging in chemsex often report difficulty separating sexual encounters from substance use, which leads to the mutual reinforcement of both addictions. This duality creates a complex clinical picture that complicates both diagnosis and treatment. The intertwining of chemical dependence with compulsive sexual behavior contributes to a feedback loop, where the use of substances becomes essential for achieving sexual gratification, while the sexual activity reinforces the craving for substances. Such interaction intensifies both the physical and psychological aspects of addiction, requiring comprehensive therapeutic strategies tailored to the unique needs of chemsex participants [71].

Chemsex can be classified as a form of mixed addiction, combining elements of behavioral addiction (e.g., compulsive sexual activity) with substance use disorder. The co-occurrence of these two components results in a shared neurobiological mechanism that reinforces the addictive cycle and impairs impulse control [3].

Another example of a mixed-type addiction is nicotine dependence, which merges the pharmacological effects of nicotine with behavioral automatisms—such as ritualistic smoking in specific emotional or social contexts. Nicotine, the main psychoactive substance in tobacco, acts on nicotinic cholinergic receptors in the brain’s mesolimbic system, stimulating dopamine release and contributing to the reinforcement of smoking behavior. Over time, this leads to a habitual and compulsive pattern, supported by overlapping dopaminergic, serotonergic, and noradrenergic dysregulations, which also play a role in mood instability and emotional regulation [72].

Similar mechanisms have been observed in chemsex-related behaviors, where the use of psychoactive substances (e.g., GHB, mephedrone, methamphetamine) enhances sexual pleasure through dopaminergic activation. This neurochemical reinforcement creates strong associations between substance use and sexual activity, making it increasingly difficult for individuals to separate one from the other. As Waszkiewicz (2022) [23] notes, chemsex involves a synergistic interaction between substance use and behavioral compulsion, which amplifies the addictive process and complicates treatment.

Furthermore, the long-term dysregulation of serotonin and norepinephrine systems—seen in both nicotine addiction and chemsex—may lead to increased anxiety, depressive symptoms, and emotional dysregulation. These shared neurobiological pathways underscore the conceptualization of chemsex as a mixed addiction, where chemical and behavioral reinforcements coalesce into a unified cycle of dependency [23,72].

Dedicated treatment programs should be developed, integrating both outpatient and inpatient therapy, with a focus on the concurrent management of substance use and sexual behavior disorders. These programs must offer a multidisciplinary approach that includes psychotherapeutic interventions (e.g., cognitive-behavioral therapy (CBT), trauma-informed therapy, sexual therapy) and pharmacological treatment when necessary. Comprehensive programs should integrate pharmacological support for substance withdrawal with psychotherapy targeting sexual behavior, emotional regulation, trauma, and underlying psychological issues [73].

In parallel, harm reduction strategies—including education on safe substance use, STI prevention, and mental health support—are critical in mitigating the immediate health risks associated with chemsex [2]. These strategies play an important role, particularly for individuals who are not yet ready to stop using substances, helping to reduce negative consequences and build trust in support systems. Despite its growing prevalence, chemsex is not currently recognized as a distinct diagnostic entity in the ICD-11 classification, which makes it difficult to systematically diagnose and treat. Its inclusion in future editions, such as the ICD-12, could facilitate better diagnostic precision and promote the development of targeted therapeutic frameworks [3].

#### 4.1.1. Diagnostic Classification of Chemsex in ICD-10 and ICD-11

According to the ICD-10 classification, substance dependence (code F19.2) is characterized by six key diagnostic criteria: (1) a strong internal drive to use the substance, (2) impaired ability to control its use, (3) continued use despite harmful consequences, (4) prioritization of substance use over other activities and responsibilities, (5) increased tolerance, and (6) withdrawal symptoms upon cessation. A diagnosis of dependence is made when three or more of these criteria are present. This framework has historically informed clinical assessments of substance-related disorders. Individuals practicing chemsex often meet several of these criteria, particularly the compulsive use of psychoactive substances in sexual contexts, the loss of control over such use, and persistence despite physical, psychological, or social harm [74].

In contrast, the ICD-11 classification (code 6C_40_) defines substance dependence more concisely, using three core features, of which at least two must be present within a 12-month period to establish a diagnosis:

- Impaired control over substance use (e.g., onset, frequency, intensity, termination).
- Increasing priority given to substance use over other activities and responsibilities.
- Physiological features, including tolerance or withdrawal, etc.

Individuals engaging in chemsex frequently fulfill at least two of these criteria—particularly the loss of control over substance use in sexual contexts and the prioritization of chemsex over other life domains—indicating a pattern consistent with substance dependence as defined by the ICD-11 [74].

Furthermore, the ICD-11 classification includes compulsive sexual behavior disorder (CSBD) under impulse control disorders (code 6C_72_). CSBD is defined as a persistent pattern of failure to control intense, repetitive sexual impulses or urges, resulting in repetitive sexual behavior. The diagnostic criteria include (1) persistent failure to resist sexual impulses despite repeated negative consequences, (2) repeated unsuccessful efforts to reduce or control sexual behavior, (3) sexual behavior becoming a central focus of the individual’s life to the detriment of other responsibilities and activities, and (4) continued engagement in sexual behavior even when it causes little or no satisfaction. The symptoms must persist for at least six months and cause significant distress or functional impairment.

Importantly, the absence of a clear ICD-10 diagnosis for CSBD has historically contributed to the underrecognition or misclassification of such behaviors in clinical settings. The introduction of CSBD in the ICD-11 (6C_72_) reflects growing recognition of the clinical significance of this phenomenon and provides a clearer framework for diagnosis and treatment [75].

In the context of chemsex, many individuals exhibit symptoms consistent with both psychoactive substance dependence and compulsive sexual behavior. The combined presence of substance-related addiction and behavioral dysregulation suggests that chemsex can be accurately classified as a mixed addiction. This dual nature requires an integrative diagnostic and therapeutic approach that addresses both components simultaneously for effective intervention and treatment [75].

#### 4.1.2. Proposal for Classification of Chemsex as a Separate Diagnostic Entity in ICD-12

Given the growing prevalence, diagnostic complexity, and public health burden associated with chemsex, we propose the introduction of a distinct diagnostic entity in the upcoming ICD-12 classification: Chemsex Use Disorder. This category would reflect the dual nature of the condition, combining features of both psychoactive substance use disorders and compulsive sexual behavior, as increasingly observed in clinical settings and addiction treatment practice [76].

The provisional diagnostic criteria for this proposed category could include the presence of at least two of the following four features, persisting over a period of at least 12 months:

- Regular and intentional use of psychoactive substances in sexual contexts.
- Inability to engage in sexual activity without the use of substances.
- Continued engagement in chemsex despite evidence of physical, psychological, or social harm.
- Persistent failure to control either substance use or sexual impulses, or both.

These proposed criteria are based on a synthesis of existing ICD-10 and ICD-11 definitions for substance dependence (codes F19.2 and 6C_40_, respectively) and compulsive sexual behavior disorder (code 6C_72_) while accounting for the specific interplay between chemical and behavioral factors observed in chemsex [73,74,75].

The justification for including chemsex as a separate diagnostic entity is multifold. First, it exhibits characteristics of both chemical and behavioral addictions, with strong comorbidity between substance dependence and dysregulated sexual behavior. Second, there is a clear causal and reinforcing relationship between the use of psychoactive substances and sexual gratification. Third, chemsex is associated with significant health risks, including increased rates of STIs, mental health deterioration, and substance-related complications [68,72]. Finally, the absence of a distinct diagnostic code hinders coherent diagnostic, therapeutic, and epidemiological strategies and limits healthcare systems’ ability to respond effectively to this growing phenomenon.

Introducing a specific ICD-12 category for chemsex-related mixed addiction could improve diagnostic accuracy, standardize clinical reporting, and support the development of targeted treatment and prevention programs. It would also facilitate international research efforts and health policy planning concerning this high-risk behavior.

### 4.2. Integrated Treatment Strategies for Mixed Addiction in Chemsex

The development of specialized treatment programs can not only increase the effectiveness of treatment but also reduce the risk of relapse and raise public awareness of chemsex as a phenomenon that requires a comprehensive approach. In addition, digital technologies such as mobile applications and telemedicine platforms can support individuals affected by chemsex by providing access to educational materials, psychological consultations, and real-time behavioral monitoring [73].

According to a report by the European Monitoring Centre for Drugs and Drug Addiction (EMCDDA), 37% of those involved in chemsex exhibit characteristics of substance dependence, and 25% report difficulty giving up drugs during sexual intercourse. Modern technologies can play a key role in countering these problems [77].

Given the dual nature of chemsex-related addiction—encompassing both psychoactive substance dependence and compulsive sexual behavior—effective treatment requires an integrated, multifaceted therapeutic approach that addresses both components simultaneously. Such strategies must be tailored to the complex clinical profile of chemsex participants and their psychosocial context.

#### 4.2.1. Cognitive–Behavioral Therapy (CBT) as a Recommended Approach in the Treatment of Chemsex

CBT is widely recognized as the gold-standard psychological intervention in the treatment of both substance use disorders and behavioral addictions, including compulsive sexual behavior [78,79,80]. In the context of chemsex, CBT’s structured, flexible, and empirically validated model is uniquely suited to address the dual nature of this condition, namely, the interaction between psychoactive substance use and dysregulated sexual behavior [81,82].

CBT focuses on understanding and modifying the dysfunctional cognitive, emotional, and behavioral patterns that sustain addictive behaviors. Key therapeutic techniques include the following:

- Cognitive restructuring—challenging irrational or harmful beliefs related to drug use, sex, identity, and control [78].
- Coping skills training—teaching strategies to handle emotional dysregulation, social rejection, loneliness, and internalized stigma [80,81].
- Trigger and craving management—identifying high-risk situations and applying personalized relapse prevention techniques [83].
- Schema therapy (ST)—focusing on early maladaptive schemas (e.g., shame, abandonment, emotional deprivation) that underlie the need for escape into altered sexual or emotional states [82,84].
- Motivational interviewing—enhancing readiness to change and reducing ambivalence, which is often high in individuals practicing chemsex [85].
- Relapse prevention—equipping patients with a toolkit of skills to prevent return to risky behaviors [83].

CBT can be delivered effectively in both outpatient and inpatient settings. In outpatient therapy, it supports behavior regulation while preserving the individual’s natural environment. In more severe cases, inpatient CBT allows for intensive daily interventions, structured routines, and close behavioral monitoring—all crucial when chemsex behavior is entrenched or co-occurs with other psychiatric conditions [78,80,81].

In the Polish therapeutic and clinical context, CBT has been extensively promoted by researchers, who emphasize its value in treating trauma, affective disorders, and impulsive–compulsive spectra [81,86]. Their work also underscores the need for trauma-informed CBT adaptations, especially in patients with coexisting developmental or identity-related vulnerabilities [87].

Recent international studies confirm the efficacy of CBT for sexualized drug use, especially when combined with harm reduction strategies, sexual health education, and HIV/pre-exposure prophylaxis (PrEP) counseling [88,89]. Research by Glynn et al. (2021) and Schmidt et al. (2023) also highlights the benefits of integrating CBT with digital therapeutic tools and peer-based interventions in reaching high-risk groups engaged in chemsex practices [90,91].

Given its strong evidence base, adaptability to comorbidities, and ability to integrate both behavioral and emotional regulation components, CBT remains the most promising and scalable psychotherapeutic method for treating Chemsex Use Disorder.

#### 4.2.2. Mobile Applications

Mobile applications have become an increasingly important tool in supporting the prevention and treatment of chemsex-related addiction. These digital solutions enable real-time behavioral tracking, provide access to psychological support, and deliver education related to sexual health and harm reduction. Their accessibility and anonymity make them particularly useful for individuals hesitant to seek traditional, in-person treatment options [91].

Modern applications often integrate multiple functions within a single platform. These include built-in tutorials on harm reduction and coping strategies, crisis communication channels with addiction specialists, calendars for monitoring substance use and emotional states, and interactive psychoeducational modules that promote self-reflection and behavior change [92].

Popular examples include Sober Grid, a social recovery network offering peer support to individuals in addiction recovery; My Quit Coach, an app that facilitates self-monitoring of habits related to substance use; and IAmSober, which tracks sobriety and helps users identify triggers while encouraging long-term commitment to recovery [29].

By reducing barriers related to stigma and increasing user engagement at early stages of recovery, mobile apps play a significant role in broadening access to care. They also complement other therapeutic methods, such as telemedicine and in-person CBT, forming part of a broader digital ecosystem designed to support patients through different phases of treatment [93].

#### 4.2.3. Telemedicine as a Support Tool

Thanks to telemedicine, it is possible to provide therapeutic support remotely, which increases the availability of help for chemsex addicts, especially in regions with limited access to medical facilities [94]. One of the key solutions is the video-consultation platform, which allows patients to contact specialists directly, significantly improving the therapeutic process [95].

An additional innovation is electronic medical records management systems, which allow for archiving the course of treatment, which is particularly important in the context of addiction therapy. Studies indicate that the use of such tools improves the effectiveness of interventions and enables faster implementation of therapeutic methods [17].

An important component of telemedicine is algorithms that analyze medical data to help identify patients most at risk of risky behavior. An example is AI used in addiction diagnosis, which predicts relapse risk based on analysis of clinical data [75].

Telemedicine is often integrated with mobile apps to build a comprehensive ecosystem of support for addicts. This combination of technologies enables behavioral monitoring, access to educational guides, and the ability to connect with therapists in real time [96].

The development of telemedicine technologies enhances the effectiveness of addiction treatment and allows therapies to be tailored to individual patients’ needs, helping to reduce the risk of relapse and improve the quality of life of addicts.

Examples of modern telemedicine platforms include the following:

- Talkspace—online therapy to support people with mental health and addiction disorders;
- Amwell—telemedicine psychiatric consultations;
- BetterHelp—access to certified inpatient therapists remotely.

The use of telemedicine allows the implementation of CBT, which can be delivered remotely, increasing the availability of treatment for people who, for various reasons, cannot attend inpatient therapy [76].

#### 4.2.4. Artificial Intelligence (AI) in Diagnostics

AI is significantly impacting the diagnosis and treatment of addiction, including chemsex. Advanced algorithms allow analysis of medical data to accurately identify patients most at risk of relapse and tailor treatment to their individual needs [95]. One of the key systems in this regard is Predict AI, which assesses the risk of relapse based on treatment history and patient behavior. A similar function is performed by Mindstrong, which uses psychological analysis and monitoring of users’ moods to detect critical moments related to addiction early on.

Increasingly, AI is also being applied to digital therapies, as shown by the example of Pear Therapeutics, where the technology assists in the treatment of addicts through interactive therapy sessions and algorithms that adjust treatment methods depending on the patient’s progress [95]. The implementation of AI in chemsex therapy has the potential to increase treatment efficacy through continuous analysis of psychological and behavioral variables, but it also brings challenges related to data privacy and the need to develop ethical standards for its use in medicine. Further research in this area is crucial to ensure the safe and effective use of AI in addiction treatment and to reduce the potential risks associated with its automated decisions [95].

## 5. Future Perspectives

One of the key challenges in the context of chemsex diagnosis is the lack of clear diagnostic criteria and its absence as a separate disease entity in the ICD-11 International Classification of Diseases. In future editions, such as the ICD-12, the inclusion of chemsex as a mixed addiction could significantly improve the diagnosis process and the implementation of more effective therapeutic strategies. Standardization of diagnostic criteria would allow better recognition of the mechanisms involved in both psychoactive substance dependence and behavioral addiction, which is characteristic of chemsex practitioners [75].

In addition, there is a significant gap in the scientific literature on chemsex among women. Research to date has focused primarily on the population of MSM, which limits a full understanding of the phenomenon in other social groups. However, there are indications of a growing interest in chemsex among women as well, especially in party and subcultural contexts where psychoactive substances are used to enhance sexual experiences [77]. Future research should include women and non-heteronormative individuals, analyzing their motivations, behavioral patterns, and health effects associated with chemsex [6].

Verifying the effectiveness of mobile apps and telemedicine platforms in monitoring users and reducing addiction relapse should also be an important research direction. Long-term cohort studies are needed to assess the impact of digital tools on improving patients’ quality of life and adherence to therapy [6].

Standardization of AI algorithms with privacy and transparency is also an indispensable part of future studies. The use of AI in analyzing the behavior of chemsex practitioners allows for the prediction of relapse and better tailoring of therapeutic interventions but requires the development of legal regulations and ethical standards to ensure adequate protection of patients [95].

At the same time, comparative studies among different populations, including women and heterosexuals, are important, which would identify specific therapeutic needs and differences in motivational mechanisms [92].

Research should also include an analysis of the impact of technology use on treatment efficacy, especially in the context of PrEP used to prevent HIV infection among chemsex practitioners [76].

Equally important is the adaptation of digital tools according to cultural context, which would allow the development of more effective interventions tailored to diverse settings. A key step will be the integration of digital technologies with psychotherapy, incorporating behavioral, environmental, and social aspects into the treatment of chemsex [77].

Future research should also lead to the implementation of chemsex as a formal diagnostic category, allowing for more precise treatments and standardization of treatment protocols in public health systems around the world [75].

## 6. Limitations

Despite advances in technology and numerous chemsex studies, there are important limitations to consider when interpreting the results. First and foremost, many studies rely on participant self-reports, which can involve reporting error and sample selection. The subjectivity of responses and the influence of social factors can lead to under- or overestimation of the magnitude of the problem [6,29].

In addition, cultural and geographic differences affect the universality of the results, as chemsex is a phenomenon strongly influenced by social context. In different regions of the world, the availability of psychoactive substances, social norms, and the level of stigmatization of those involved in chemsex can significantly affect how the phenomenon is perceived and studied [93,97].

Another limitation is the high demand for resources—both financial and technological—in implementing advanced telemedicine and AI systems. The implementation of modern technologies requires adequate infrastructure and access to skilled professionals, which may reduce their availability in less-resourced regions. In addition, the effectiveness of AI tools in analyzing patient behavior depends on the quality of available data, which is often incomplete or subject to errors [94].

Ultimately, ethical aspects, including data protection and transparency of algorithms, remain controversial and require the development of international standards. The use of AI in the diagnosis and treatment of chemsex raises questions about patient privacy, accountability for decisions made by AI systems, and equal access to modern treatments.

In the future, further research will need to be conducted on the effectiveness of the technology in the treatment of chemsex, and regulations will need to be developed to ensure patient safety and transparency in the methods used.

## 7. Discussion

In light of the literature review and current clinical trends, the phenomenon of chemsex appears as a diagnostic and therapeutic challenge that has not yet been adequately addressed within the framework of current nosological classifications. A key problem is the lack of clear assignment of this phenomenon in the International Classification of Diseases ICD-11, which limits the possibilities for diagnosis and therapeutic interventions [37,98]. In light of the available empirical data and analyses of clinical cases, it seems reasonable to consider locating chemsex as a dual addiction entity—combining elements of psychoactive substance addiction and compulsive sexual behavior [17].

Epidemiological data and clinical reports suggest that the most commonly used substances in the context of chemsex worldwide are methamphetamine, GHB/GBL, and mephedrone. Their popularity varies by region—methamphetamine dominates in North America and Southeast Asia, while GHB/GBL and mephedrone are more frequently used in Europe [29,31].

Among these substances, methamphetamine is considered particularly dangerous due to its high addictive potential, risk of psychosis, and neurotoxicity [28,99]. GHB and its precursors also pose serious risks, especially regarding respiratory depression, loss of consciousness, and accidental overdose—particularly when combined with alcohol or benzodiazepines [61].

Synthetic opioids such as fentanyl, although less frequently used in chemsex, are extremely toxic even at very low doses and are responsible for an increasing number of deaths in some regions [61]. These findings highlight the importance of substance-specific prevention strategies and the need to implement harm reduction interventions.

The sociocultural context remains an important factor influencing the development of chemsex. Chemsex practices have not only a pharmacological dimension but also a social one—they enable identity exploration, escape from normative tension, and temporary relief from existential stress. Minority stress mechanisms, internal homophobia, and lack of social acceptance promote risky sexual behavior under the influence of psychoactive substances [16,93].

Studies indicate that people who engage in chemsex often look to it as a way to increase self-confidence, overcome fear of rejection, or obtain temporary relief from emotional tensions. The neurobiological underpinnings of this phenomenon must also be taken into account—the synergistic combination of psychoactive substance addiction with a behavioral addiction such as sex addiction significantly complicates the treatment and diagnosis process [93].

Against the background of classical therapies, modern forms of treatment—especially those using digital technologies—are now gaining particular importance [100]. An analysis of the literature and existing technological implementations indicates the growing potential of modern methods of prevention and treatment of chemsex-related addiction [35]. On the one hand, mobile apps, telemedicine, and AI-based tools enable access to rapid, personalized care, reducing the geographic barriers and stigma that often limit patients’ contact with traditional health care providers [54,92]. On the other hand, there are significant challenges, such as ensuring full protection of personal data and guaranteeing equality of access regardless of economic or geographic conditions [101].

In addition, the use of AI in predicting recurrence and analyzing patient behavior patterns opens up new opportunities for personalizing treatment. Predictive models based on behavioral data can, over time, assist clinicians in making more accurate therapeutic decisions, assuming digital ethics and patient data accountability are maintained [102].

In parallel with technological developments, new substances used in chemsex are emerging, such as fentanyl, kratom, and psychedelics, whose effects can lead to unpredictable bodily reactions and significant health risks. It is therefore necessary not only to implement modern forms of therapy but also to update clinical knowledge of their effects and monitor their prevalence.

Effective interventions should combine elements of CBT, pharmacotherapy, and technological support. It is also recommended that dedicated treatment programs be developed for different demographic groups—not only for MSM but also for women and heterosexuals who are also beginning to experience chemsex-related problems. Additionally, an important direction for future work is to include the topic of chemsex in the diagnostic classifications of the ICD-11, which would allow for a clear diagnosis and the development of standardized therapeutic strategies. Currently, the lack of formal diagnosis results in difficulties in accessing help, which can exacerbate the marginalization of those affected.

The introduction of dedicated treatment programs, both outpatient and inpatient, can significantly improve the effectiveness of chemsex treatment and reduce the risk of relapse.

## 8. Conclusions

Chemsex is a complex phenomenon that requires an interdisciplinary approach to both diagnosis and treatment. With the rapid development of digital technologies, there are new opportunities in addiction prevention and treatment but also challenges in implementation. The use of mobile applications, telemedicine, and AI-based systems offers potential not only in increasing accessibility to therapeutic support but also in personalizing interventions, which can translate into reduced relapse rates and improved mental and physical health for patients The introduction of CBT therapy in both outpatient and inpatient formats is a key component of effective chemsex treatment. This therapy allows for the identification of destructive thought patterns and the development of coping strategies for dealing with impulses to use psychoactive substances in a sexual context. Combined with modern technology, such as mobile apps that support self-control and AI systems that analyze behavioral patterns, a more comprehensive treatment model is possible

Despite existing limitations related to privacy, technology standardization, and cultural differences, further research—especially that based on long-term cohort studies—could contribute to the development of more effective prevention strategies. The inclusion of chemsex as a separate diagnostic category in future editions of classifications such as the ICD-12 could significantly facilitate the process of diagnosis and the implementation of dedicated therapies.

With the growing health risks of chemsex, the integration of modern technologies remains one of the most important elements of a future public health strategy. Combining innovative therapies with digital diagnostic tools can not only increase the effectiveness of treatment but also improve the quality of life of those affected.

## Figures and Tables

**Table 1:** Inclusion and exclusion criteria for studies included in the review.

Inclusion Criteria	Exclusion Criteria
Publications from 2015–2025 providing up-to-date data on chemsex and its health consequences.	Publications not subject to peer review and opinion articles without empirical data.
Studies on psychosocial aspects of chemsex, therapeutic technologies, and effectiveness of modern treatment methods.	Studies focusing only on general social aspects of chemsex, without reference to health effects.
Studies analyzing the interaction of psychoactive substances with mental and sexual health.	In vitro laboratory work not providing direct evidence of chemsex effects on the human body.
Articles describing therapeutic strategies, including telemedicine and AI tools in monitoring user behavior patterns.	Publications with low methodological transparency and high risk of bias.

**Table 2:** Epidemiological characteristics of chemsex participants by gender identity.

Gender Identity	Main Characteristics	Risks/Outcomes	References
Men (MSM)	High prevalence; frequent use of methamphetamine, mephedrone, GHB/GBL; slamsex practices	High risk of HIV and STIs; frequent use of dating apps for chemsex organization	[6,12,13,16,18,25,28,29,30,31,32]
Women	Lower prevalence; often in recreational/club contexts; underrepresented in studies	Risk of sexual assault under the influence; limited epidemiological data	[6,33,34,35]
Transgender individuals *	Higher vulnerability; frequent polysubstance use	Severe health and psychosocial consequences; compounded by stigma and minority stress	[30,36,37]

* Further discussed in Section 3.2.2.

**Table 4:** Characteristics of new substances used in chemsex. Based on [57,61,62,63,65].

Substance	Action	Potential Risks
Psychedelics (LSD, psilocybin, DMT, mescaline)	Change in perception, intensification of sensations, introspection	Anxiety, hallucinations
Sodium nitroprusside	Strong vasodilation of blood vessels	Dangerous drops in blood pressure, risk of fainting
α-PVP (“Flakka”)	Euphoria, heightened alertness, insomnia, psychomotor agitation, greater resistance to pain	Increase in aggressiveness, overexertion, anxiety, heart rhythm disturbances
UR-144	Relaxing, mood-enhancing	Loss of consciousness, dependence, coordination disorders

## Abbreviations

The following abbreviations are used in this manuscript:

ADHD	attention deficit hyperactivity disorder
AI	artificial intelligence
ART	antiretroviral therapy
CBT	cognitive behavioral therapy
CSBD	compulsive sexual behavior disorder
EMCDDA	European Monitoring Centre for Drugs and Drug Addiction
GHB	γ hydroxybutyric acid
GBL	γ butyrolactone
HIV	human immunodeficiency virus
HPV	human papillomavirus
MDMA	3,4 methylenedioxymethamphetamine
MSM	men who have sex with men
NPS	new psychoactive substance
PDE	5 phosphodiesterase type 5
PrEP	pre-exposure prophylaxis of HIV infection
ST	schema therapy
STIs	sexually transmitted infections
SUD	substance use disorder
αPVP	α-pyrrolidinopentiophenone
